# The Role of Health in the Technology Acceptance Model Among Low-Income Asian American Older Adults: Cross-Sectional Survey Analysis

**DOI:** 10.2196/57009

**Published:** 2024-12-03

**Authors:** Pauline DeLange Martinez, Daniel Tancredi, Misha Pavel, Lorena Garcia, Heather M Young

**Affiliations:** 1 Betty Irene Moore School of Nursing University of California, Davis Sacramento, CA United States; 2 Department of Pediatrics University of California, Davis Sacramento, CA United States; 3 Khoury College of Computer Science Northeastern University Boston, MA United States; 4 Department of Public Health Sciences University of California, Davis Sacramento, CA United States

**Keywords:** aged, older adults, Asian American, immigrant, vulnerable populations, internet, information and communications technology, ICT, digital divide, technology acceptance model, mobile phone

## Abstract

**Background:**

Self-rated health is associated with information and communications technology (ICT) use among older adults. Non–US born, older Asian American individuals are more inclined to rate their health as fair or poor compared to individuals from other racial and ethnic backgrounds. This population is also less likely to use ICTs as compared to White older Americans. Furthermore, cognitive decline may impact technology acceptance. In a previous adaptation of the technology acceptance model for low-income, Asian American older adults, perceived usefulness (PU), perceived ease of use (PEOU), age, educational attainment, ethnicity, and English proficiency were significant predictors of ICT use. However, the association between health and technology acceptance has not been explored among Asian American older adults.

**Objective:**

This study examined the role of self-rated health and subjective cognitive decline in the acceptance and use of ICTs among low-income, Asian American older adults.

**Methods:**

This cross-sectional survey included Asian American individuals aged ≥62 years living in affordable housing for older adults (N=392). Using hierarchical multiple regression, we explored the association between self-rated health and ICT use and technology acceptance model mediators (PU and PEOU) while adjusting for demographics, English proficiency, and subjective cognitive decline. Contrast statements were used to estimate contrasts of interest. To further examine the separate and joint association between age and subjective cognitive decline and the dependent variables, we examined scatterplots with locally estimated scatterplot smoothing lines, revealing that the relationship between subjective cognitive decline and ICT use varied in 3 age segments, which led to updating our analysis to estimate differences in ICT use among age categories with and without subjective cognitive decline.

**Results:**

Self-rated health was not significantly associated with ICT use (β=.087; *P*=.13), PU (β=.106; *P*=.10), or PEOU (β=.062; *P*=.31). However, the interaction terms of subjective cognitive decline and age significantly improved the model fit for ICT use (Δ*R*^2^=0.011; *P*=.04). In reviewing scatterplots, we determined that, in the youngest age group (62-74 years), ICT use increased with subjective cognitive decline, whereas in the older age groups (75-84 and ≥85 years), ICT use decreased with subjective cognitive decline, more so in the oldest age category. Through regression analysis, among participants with subjective cognitive decline, ICT use significantly decreased in the middle and older age groups as compared to the youngest age group. However, among participants without subjective cognitive decline, the difference in use among age groups was not significant.

**Conclusions:**

This study contributes to the understanding of the complex relationship between health and ICT acceptance among low-income, Asian American older adults and suggests the need for tailored interventions to promote digital engagement and quality of life for this population.

## Introduction

### Background

Asian Americans are the fastest-growing ethnic group aged ≥65 years in the United States, with 85% of this group being non–US born [1-4]. Despite their rapid population growth, Asian American older adults remain under-studied and under-served, attributed in part to their heterogeneity, encompassing >40 ethnic subgroups characterized by diverse languages, cultural backgrounds, and immigration histories [1,2,5-7]. Asian American older adults are less likely to use information and communications technologies (ICTs) such as smartphones, tablets, computers, the internet, social media, or other applications as compared to White older Americans [8,9]. Intersectional factors such as socioeconomic status and English proficiency play a significant role in diminishing the likelihood of ICT use among this population [8-10]. According to data from the California Health Interview Survey (CHIS), non–US born Asian American older adults are more inclined to rate their health as fair or poor compared to individuals from other racial and ethnic backgrounds [11]. While there is a significant association between self-rated health and ICT use among older adults [12-15], this association has not been explored among Asian American older adults.

Self-rated health is a subjective measure that encompasses multiple dimensions of an individual’s well-being, including physical, mental, social, and functional aspects. Self-rated health is influenced by personal and cultural beliefs as well as health behaviors [16]. Self-rated health has an independent effect on health outcomes even when adjusting for other measures of medical, physical, cognitive, emotional, and social health [17]. Among Asian American individuals, self-rated health is influenced by factors such as gender; age; and level of acculturation, including English proficiency [18].

Poor self-rated health among Asian American older adults is also closely linked to mental well-being. Analysis of survey data from a large sample of Korean American and Chinese American older adults collected from 2011 to 2019 uncovered significant and positive associations among poor self-rated health, depression, number of chronic diseases diagnosed, and functional disabilities [19]. Furthermore, CHIS data revealed that Asian American older adults with limited English proficiency (LEP) were 3 times more likely to have a perceived mental health need as compared to their English-speaking counterparts [20]. According to the National Asian Pacific Center on Aging, >60% of Asian American individuals aged ≥65 years have LEP, and these individuals are more likely to rate their health as poor [1,20]. Among Asian American older adults, poor self-rated health is attributed to a variety of factors, including low educational attainment, low health literacy, racism and discrimination, disparities in quality of care accumulated over time, and delayed use of health care [11]. Self-rated health among Asian American older adults may be further impacted by challenges such as living far from relatives and experiencing cultural differences and limited social networks in their new environment [1,21,22].

Asian American older adults have higher poverty rates (9.3%) than the general older adult population in the United States (8.9%) [23]. Low-income older adults are more likely to rate their health as poor and report lower ICT use [24,25]. In an analysis of the CHIS, the combination of Asian ethnicity and low income had an interactive, negative effect on ICT use [10]. In fact, Asian American older adults in the lowest income category were 14 times less likely to use the internet for health information compared to non-Hispanic White older adults in the highest income category [10]. More research is needed to understand the factors that impact ICT adoption among low-income, ethnic minority older adults to inform interventions to address this digital divide.

### Theoretical Framework

The technology acceptance model (TAM) is a widely accepted framework used to understand the adoption of new technologies [26]. The model suggests that attitudes toward technology, including perceived usefulness (PU) and perceived ease of use (PEOU), significantly influence an individual’s intention to use and actual use of technology.

Nayak et al [14] and Chen and Chan [15] each independently adapted the TAM, adding self-rated health as a covariate predicting ICT use among older adults. In a study of adults aged ≥60 years in the United Kingdom, poor self-rated health was significantly, positively associated with hours per week spent using the internet when adjusting for attitudes toward technology, including PU and PEOU, and demographics [14]. Similarly, in a study of adults aged ≥55 years in Hong Kong, poor self-rated health was positively associated with use of gerontechnologies, a broad term encompassing ICTs, housing and daily living technology, health technology, and education and recreation technologies, when adjusting for attitudes toward technology and demographics. In this study, other health variables were incorporated as covariates, including number of health conditions, physical functioning, cognitive ability, social relationships, and attitude toward life and satisfaction [15].

While Nayak et al [14] and Chen and Chan [15] reported an inverse relationship between self-rated health and technology use, other studies have found them to be positively associated. Werner et al [12] conducted a cross-sectional survey analysis of ethnically and income-diverse adults aged ≥60 years in the United States, demonstrating that better ratings of general physical health and role-related emotional health were independently and positively associated with email and computer use, respectively, when adjusting for demographic factors. In another cross-sectional survey analysis engaging adults aged ≥80 years, Sims et al [13] found that participants with better self-rated health were more likely to report using a greater variety of ICT devices and applications.

Overall, studies support a significant association between self-rated health and ICT use among older adults. However, some have found this relationship to be positive [12,13], whereas others have found it to be negative [14,15]. This association has not been explored specifically among older Asian American individuals, who are more likely to rate their health as fair or poor as compared to older adults of other ethnicities [11].

### Cognitive Ability and ICT Use

Beyond self-rated health, cognitive ability is another important predictor of ICT use. When adjusting for self-rated health and demographic factors, cognitive ability was significantly, positively associated with gerontechnology use and ICT use [15]. This relationship has been confirmed in multiple studies. For example, in a cross-sectional study of adults aged ≥65 years, subjective cognitive decline was significantly negatively associated with frequency of ICT use even when adjusting for age, educational attainment, and depression [27]. In addition, in an analysis of the National Health and Aging Trends Study, self-reported memory problems were associated with decreased email, SMS text message, and internet use even when adjusting for age, sex, race and ethnicity, educational attainment, marital status, multimorbidity, and self-rated health [28]. In this study, we examine the influence of self-rated health on ICT acceptance among Asian American older adults adjusting for subjective cognitive decline.

### Research Design

This cross-sectional study examined the relationships among self-rated health, attitudinal factors (PU and PEOU), and ICT use among low-income, Asian American older adults.

**Figure 1:**
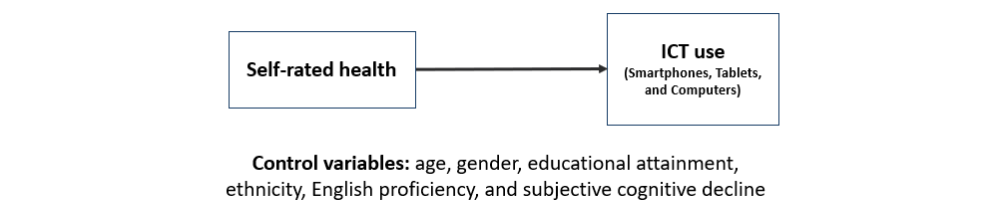
Model examining the association between self-rated health and information and communications technology (ICT) use among Asian American older adults in low-income housing to test hypothesis 1.

**Figure 2:**
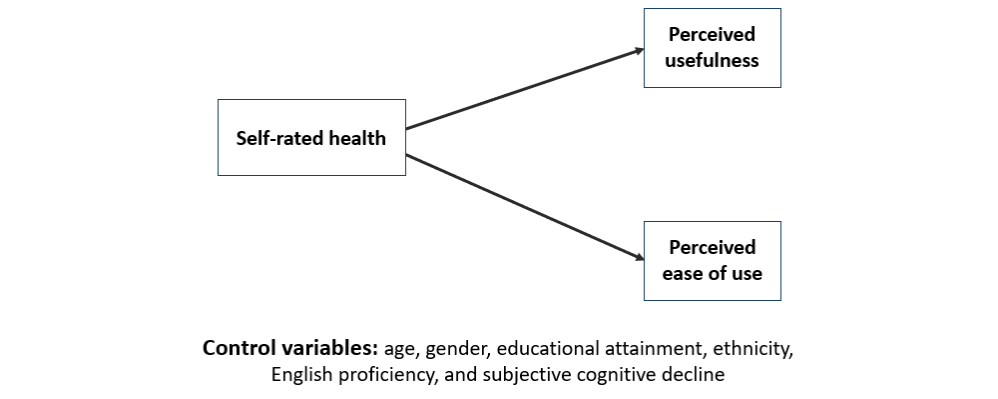
Model examining the association among self-rated health, perceived usefulness, and perceived ease of use among Asian American older adults in low-income housing to test hypothesis 2. For each dependent variable shown on the right, a separate regression analysis was run.

**Figure 3:**
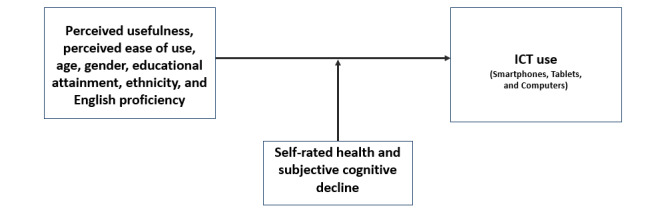
Model examining self-rated health and subjective cognitive decline as potential moderating or effect-modifying factors among perceived usefulness, perceived ease of use, demographic factors, and information and communications technology (ICT) use among Asian American older adults in low-income housing to test hypothesis 3.

In this study, we tested three hypotheses:

1. Self-rated health will be significantly, positively associated with ICT use when adjusting for age, gender, educational attainment, ethnicity, English proficiency, and subjective cognitive decline (hypothesis 1, Figure 1).
2. Self-rated health will be significantly, positively associated with PU and PEOU when adjusting for age, gender, educational attainment, ethnicity, English proficiency, and subjective cognitive decline (hypothesis 2, Figure 2).
3. Adding health variables (self-rated health and subjective cognitive decline) as main effects or interaction terms to the TAM will significantly improve model fit (hypothesis 3, Figure 3).

In examining hypotheses 1 and 2, we investigated PU and PEOU as potential mediators of the relationship between self-rated health and ICT use. Hypothesis 3 examines self-rated health and subjective cognitive decline as potential moderating or effect-modifying factors among PU; PEOU; demographic factors; and our dependent variable, ICT use. The exploration of effect modification is novel, but we felt that it was necessary to apply this model to an older adult population. This research builds on previous work [29].

## Methods

### Data and Sample

This study was a secondary data analysis of the Lighthouse for Older Adults program. The Lighthouse for Older Adults program provided high-speed broadband access, ICT devices, and a series of digital literacy trainings to residents living in 8 affordable housing communities for older adults located across California. All residents (N=1050) were invited to participate. To recruit participants, on-site staff distributed flyers about the Lighthouse program, set up informational tables in high traffic areas of the community, and held social events where residents could test out a variety of ICT devices to inform device selection for the project. All Lighthouse participants were required to complete pre- and postsurveys as well as attend at least one digital literacy training course. All participants were of low income; to qualify for affordable housing for older adults, residents’ incomes must be <80% of the local area median income. The surveys were evidence based, and pilot-tested with residents and staff in 5 languages. The surveys were translated and then back translated by professional translators. The final surveys were self-administered in the residents’ preferred language (eg, Korean, Chinese, Vietnamese, Japanese, Hmong, and English), with staff available to assist as needed.

This study is a cross-sectional survey analysis of the presurveys from the Lighthouse program. Participants were included in this study if they were aged ≥62 years and identified as being of Asian ethnicity. We excluded participants who were missing survey responses to items incorporated into the outcome variable (ICT use). The final dataset included 392 participants.

### Ethical Considerations

On the basis of the Human Research Protection-210 Determination Request, the University of California, Davis, Institutional Review Board determined that this research was exempt as it did not directly involve human participants and used deidentified secondary data (1938286-1). The original informed consent allowed for secondary analysis without additional consent, and the survey results were deidentified to maintain the privacy and confidentiality of the participants. As compensation for participating in the Lighthouse program, participants received a free ICT device along with a series of in-language digital literacy training classes. A description of Lighthouse program data collection and measures has been published previously [29].

### Measures

#### Overview

The operationalization of ICT use, attitudinal variables (PU and PEOU), and demographic variables (age, gender, educational attainment, ethnicity, and English proficiency) has been previously reported [29] and is described in Table 1. We opted to combine smartphone, computer, and tablet use into a single outcome, referred to as ICT use, given that older adults often use these devices interchangeably for various tasks [30].

**Table 1:** Operationalization of outcome and mediator variables.

Variable and measures		Response options	Internal consistency, Cronbach α
**ICT^a^ use**			
	How often do you use a desktop or laptop computer? How often do you use a tablet or iPad? How often do you use a smartphone (iPhone or Android)? How long have you been using technology, such as a computer, laptop, tablet, or smartphone?	0 (“never” or “I do not own”), 1 (“once or less than once per week”), 2 (“2 to 4 times per week”), or 3 (“about once per day”) 0 (“I’ve never used these”), 1 (“less than 1 year”), 2 (“1-2 years”), or 3 (“more than 2 years”)	0.74
**Perceived usefulness**			
	Technology helps me be connected with family and friends. Technology helps me learn new information and skills.	Scale of 1 (“strongly disagree”) to 4 (“strongly agree”)	—^b^
**Perceived ease of use**			
	I feel comfortable with technology (reverse scored). Technology makes me nervous. I don’t feel confident about my ability to use technology. Technology is confusing. I feel apprehensive about using technology. I hesitate to use technology for fear of making mistakes I cannot correct.	Scale of 1 (“strongly agree”) to 4 (“strongly disagree”)	0.89

^a^ICT: information and communications technology.

^b^Not applicable.

The operationalization of ICT use was assessed by 4 researchers with expertise in survey methodology with older adults, and the data were reviewed by a biostatistician and a nurse researcher. Examination of a predicted probability plot indicated normal distributions of residuals aligning closely with the plot’s diagonal line. Furthermore, a scatterplot of residuals showed no obvious pattern, with points equally distributed above and below 0 on the x-axis and to the left and right of 0 on the y-axis.

#### Self-Rated Health

Self-rated health was operationalized as the average of 2 survey items: “In general, how would you rate your physical health?” and “In general, how would you rate your emotional health?” Responses were scored on a scale from 1 to 5, ranging from poor to excellent. These items were adapted from the validated Patient-Reported Outcomes Measurement Information System global physical health scale [31].

#### Subjective Cognitive Decline

We included subjective cognitive decline as a covariate; this was measured using 1 item: “During the past 12 months, have you experienced confusion or changes in memory that is happening more often or is getting worse?” Response options were binary. This item came from the cognitive decline module of the validated Behavioral Risk Factor Surveillance System [32]. On the basis of feedback from staff at affordable housing for older adults, the item was slightly adapted to increase cultural sensitivity and minimize participant stigma by modifying the phrase “memory loss” to “changes in memory.”

### Analytic Strategy

First, we computed descriptive statistics for demographics, ICT use, self-rated health, and subjective cognitive decline. Next, we used Pearson correlation analysis to examine relationships among all dependent and independent variables.

To test hypotheses 1 and 2, we conducted linear regression using self-rated health as the independent variable. To test hypothesis 1, ICT use was examined as the dependent variable; to test hypothesis 2, PU and PEOU were examined as dependent variables. We adjusted for age, gender, educational attainment, English proficiency, ethnicity, and subjective cognitive decline in all models.

To test hypothesis 3, we conducted hierarchical multiple regression using stepwise blocks [33]. Demographic variables, PU, and PEOU were added in the first step (model 1). Health variables (self-rated health and subjective cognitive decline) were added in the second step (model 2). Finally, in model 3, interaction terms were included in a forward stepwise regression. A total of 12 interaction terms were tested, including all pairwise combinations of health variables (self-rated health and subjective cognitive decline) with demographic and attitudinal variables (age, gender, educational attainment, English proficiency, PU, and PEOU). At each step, goodness of fit (adjusted *R*^2^) was assessed, that is, the percentage of variability in the dependent variable that could be accounted for by the predictors. With each new set of terms, the change in *R*^2^ was calculated to quantify the change in the predictive power. In case interactions were found, contrast statements were used to estimate contrasts of interest.

To further examine the separate and joint association between age and subjective cognitive decline and ICT use, we examined scatterplots with locally estimated scatterplot smoothing lines with age as the predictor. In total, 2 scatterplots were produced, including one with a single scatterplot of data from all participants and another set stratified by presence of subjective cognitive decline. From there, we determined that the use of 3 major age categories captured the relationships well (ages of 62-74, 75-84, and ≥85 years). This led to us updating our analysis of hypothesis 3 and the associated contrast statements to estimate differences in ICT use among the age categories with and without subjective cognitive decline. For all analyses, the α level for testing significance was set to .05. All data analyses were conducted using SPSS Statistics (version 28; IBM Corp).

## Results

### Overview

Participant demographics are described in Table 2. For the sample of 392 participants, the average age was 79.1 (SD 6.95) years.

In examining the 2 health variables, most participants (270/392, 68.9%) reported fair or poor self-rated health, and 27.9% (106/380) of the participants reported subjective cognitive decline. A total of 3.1% (12/392) of the participants declined to respond to the survey question about subjective cognitive decline. The prevalence of fair or poor self-rated health and subjective cognitive decline is detailed by demographic group in Table 3. Next, we used correlation analysis to further examine relationships among ICT use, PU, PEOU, health, and demographic characteristics (Table 4).

**Table 2:** Participant demographics (N=392)^a^.

		Participants, n (%)
**Age (y)**		
	62-74	105 (27.6)
	75-84	192 (50)
	85-97	84 (22)
**Gender**		
	Women	266 (68.2)
	Men	124 (31.8)
**Ethnicity**		
	Asian: other (eg, Japanese or Hmong)	14 (3.6)
	Chinese	73 (18.6)
	Filipino	12 (3.1)
	Korean	274 (69.9)
	Vietnamese	19 (4.8)
**English proficiency**		
	Very good	8 (2.1)
	Good	63 (16.3)
	Not good	198 (51.3)
	None at all	117 (30.3)
**Educational attainment**		
	Never attended school	22 (5.9)
	Some high school	133 (35.4)
	Completed high school or GED^b^	76 (20.2)
	Some college	62 (16.5)
	College degree	60 (16)
	Graduate degree	23 (6.1)
**Years of experience using ICTs^c^**		
	>2	227 (57.9)
	1-2	45 (11.5)
	<1	30 (7.7)
	I have never used these	90 (23)
**Computer use**		
	Approximately once per day	89 (22.7)
	2-4 times per week	30 (7.7)
	Once or less than once per week	27 (6.9)
	Never	246 (62.8)
**Tablet use**		
	Approximately once per day	106 (27)
	2-4 times per week	35 (8.9)
	Once or less than once per week	20 (5.1)
	Never	231 (58.9)
**Smartphone use**		
	Approximately once per day	243 (62)
	2-4 times per week	40 (10.2)
	Once or less than once per week	17 (4.3)
	Never	92 (23.5)
**Number of ICT device types used (computer, tablet, or smartphone)**		
	No devices	78 (19.9)
	1 type	129 (32.9)
	2 types	77 (19.6)
	3 types	108 (27.6)

^a^Missing data: 11 participants did not report their age, 2 participants did not report their gender, 6 participants did not report English proficiency, and 16 participants did not report their educational attainment.

^b^GED: General Educational Development.

^c^ICT: information and communications technology.

**Table 3:** Prevalence of fair or poor self-rated health and subjective cognitive decline among Asian American older adults in low-income housing by demographic group.

		Fair or poor self-rated health, n/N (%)	Subjective cognitive decline, n/N (%)
All participants		270/392 (68.9)	106/380 (27.9)
**Age (y)**			
	62-74	63/105 (60)	16/105 (15.2)
	75-84	131/192 (68.2)	57/186 (30.6)
	85-97	66/84 (78.6)	30/81 (37)
**Gender**			
	Men	74/124 (59.7)	25/121 (20.7)
	Women	194/266 (72.9)	81/257 (31.5)
**Educational attainment**			
	High school degree or lower	173/231 (74.9)	63/226 (27.9)
	Some college or higher	83/145 (57.2)	39/139 (28.1)
**English proficiency**			
	Limited or no English	240/315 (76.2)	93/305 (30.5)
	Speak English well or very well	27/71 (38)	11/69 (15.9)
**Ethnicity**			
	Asian: other	9/14 (64.3)	2/12 (16.7)
	Chinese	56/73 (76.7)	21/70 (30)
	Filipino	4/12 (33.3)	1/11 (9.1)
	Korean	190/274 (69.3)	76/268 (28.4)
	Vietnamese	11/19 (57.9)	6/19 (31.6)

**Table 4:** Correlation analysis (Pearson r and 2-tailed *P* value) among the research variables (N=392).

		ICT^a^ use	PU^b^	PEOU^c^	Self-rated health	Subjective cognitive decline	Age	Gender (men)	Educational attainment	English proficiency
**ICT use**										
	*r*	1	0.218^d^	0.327^d^	0.248^d^	−0.121^d^	−0.225^d^	0.187^d^	0.321^d^	0.260^d^
	*P* value	—^e^	<.001	<.001	<.001	.02	<.001	<.001	<.001	<.001
**PU**										
	*r*	0.218^d^	1	0.139^d^	0.141^d^	−0.055	−0.087	0.019	0.129^d^	0.064
	*P* value	<.001	—	.007	.007	.30	.10	.72	.01	.22
**PEOU**										
	*R*	0.327^d^	0.139^d^	1	0.191^d^	−0.126^d^	−0.141^d^	0.157^d^	0.186^d^	0.240^d^
	*P* value	<.001	.007	—	<.001	.02	.006	.002	<.001	<.001
**Self-rated health**										
	*r*	0.248^d^	0.141^d^	0.191^d^	1	−0.327^d^	−0.225^d^	0.117^d^	0.297^d^	0.406^d^
	*P* value	<.001	.007	<.001	—	<.001	<.001	.02	<.001	<.001
**Subjective cognitive decline**										
	*r*	−0.121^d^	−0.055	−0.126^d^	−0.327^d^	1	0.198^d^	−0.113^d^	−0.025	−0.183^d^
	*P* value	.02	.30	.02	<.001	—	<.001	.03	.64	<.001
**Age**										
	*r*	−0.225^d^	−0.087	−0.141^d^	−0.225^d^	0.198^d^	1	−0.009	−0.106^d^	−0.194^d^
	*P* value	<.001	.10	.006	<.001	<.001	—	.86	.04	<.001
**Gender (men)**										
	*r*	0.187^d^	0.019	0.157^d^	0.117^d^	−0.113^d^	−0.009	1	0.331^d^	0.189^d^
	*P* value	<.001	.72	.002	.02	.03	.86	—	<.001	<.001
**Educational attainment**										
	*r*	0.321^d^	0.129^d^	0.186^d^	0.297^d^	−0.025	−0.106^d^	0.331^d^	1	0.403^d^
	*P* value	<.001	.01	<.001	<.001	.64	.04	<.001	—	<.001
**English proficiency**										
	*r*	0.260^d^	0.064	0.240^d^	0.406^d^	−0.183^d^	−0.194^d^	0.189^d^	0.403^d^	1
	*P* value	<.001	.22	<.001	<.001	<.001	<.001	<.001	<.001	—
**Korean**										
	*r*	−0.095	−0.203^d^	−0.131^d^	−0.063	0.016	0.003	0.014	−0.015	0.020
	*P* value	.06	<.001	.01	.21	.76	.96	.78	.77	.70
**Chinese**										
	*r*	0.051	0.171^d^	0.058	−0.056	0.022	0.102^d^	0.030	−0.048	−0.280^d^
	*P* value	.31	.001	.25	.27	.67	.047	.56	.36	<.001
**Vietnamese**										
	*r*	0.100^d^	0.049	−0.014	0.007	0.019	−0.113^d^	−0.001	0.002	0.079
	*P* value	.049	.35	.79	.89	.71	.03	.98	.97	.12
**Filipino**										
	*R*	0.104^d^	0.091	0.220^d^	0.252^d^	−0.072	−0.077	−0.090	0.172^d^	0.329^d^
	*P* value	.04	.08	<.001	<.001	.16	.13	.08	.001	<.001
**Asian—other**										
	*r*	−0.084	−0.002	0.011	0.032	−0.045	−0.012	−0.013	−0.028	0.142^d^
	*P* value	.10	.97	.83	.52	.38	.81	.79	.59	.005

^a^ICT: information and communications technology.

^b^PU: perceived usefulness.

^c^PEOU: perceived ease of use.

^d^The correlation is significant at a significance level of .01 (2-tailed).

^e^Not applicable.

### Testing Hypothesis 1: Linear Regression Examining the Association Between Self-Rated Health and ICT Use

Self-rated health was not a significant predictor of ICT use when adjusting for age, gender, educational attainment, English proficiency, ethnicity, and subjective cognitive decline; the standardized coefficient of self-rated health was 0.087 (95% CI –0.131 to 0.978; *P*=.13; Table 5). Therefore, our findings do not support hypothesis 1 (self-rated health will be significantly, positively associated with ICT use).

**Table 5:** Linear regression to examine the predictive value of self-rated health on information and communications technology (ICT) use among Asian American older adults in low-income housing adjusting for control variables (N=392)^a^.

Independent variable	ICT use	
	β^b^ (95% CI)	*P* value
Age	–.154 (–0.137 to –0.029)	.003
Gender (men)	.070 (–0.281 to 1.412)	.19
Educational attainment	.214 (0.276-0.903)	<.001
English proficiency	.110 (–0.073 to 1.218)	.08
Korean	Reference group	Reference group
Chinese	.120 (0.184-2.198)	.02
Vietnamese	.087 (–0.176 to 3.102)	.08
Filipino	−.001 (–2.327 to 2.296)	.99
Asian—other	−.083 (–4.132 to .331)	.10
Self-rated health	.087 (–.131 to .978)	.13
Subjective cognitive decline	−.055 (–1.346 to .413)	.30

^a^β values are standardized regression coefficients.

^b^Adjusted *R*^2^=0.171.

### Testing Hypothesis 2: Linear Regression Examining Outcomes of PU and PEOU

Using multiple regression, we determined that self-rated health was not a significant predictor of PU (β=.106, 95% CI −0.022 to 0.274; *P*=.10) or PEOU (β=.062, 95% CI −0.073 to 0.231; *P*=.31) when adjusting for age, gender, educational attainment, English proficiency, ethnicity, and subjective cognitive decline (Table 6). Our findings do not support hypothesis 2 (self-rated health will be significantly, positively associated with PU and PEOU).

**Table 6:** Linear regression with perceived usefulness (PU) and perceived ease of use (PEOU) as dependent variables among Asian American older adults in low-income housing (N=392)^a^.

Independent variable	PU			PEOU	
	β^b^ (95% CI)	*P* value	β^c^ (95% CI)		*P* value
Age	−.049 (–0.021 to 0.008)	.38	−.059 (–0.023 to 0.006)		.27
Gender (men)	−.016 (–0.258 to 0.196)	.79	.120 (0.022-0.489)		.03
Educational attainment	.063 (–0.041 to 0.129)	.31	.037 (–0.060 to 0.114)		.54
English proficiency	.006 (–0.166 to 0.181)	.93	.120 (–0.012 to 0.343)		.07
Chinese	Reference group	Reference group	Reference group		Reference group
Korean	−.259 (–0.804 to –0.263)	<.001	−.159 (–0.629 to –0.075)		.01
Vietnamese	−.055 (–0.713 to 0.267)	.37	−.094 (–0.921 to 0.091)		.11
Filipino	.026 (–0.535 to 0.810)	.69	.151 (0.173 to 1.558)		.01
Asian: other	−.046 (–0.936 to 0.394)	.42	−.032 (–0.849 to 0.470)		.57
Self-rated health	.106 (–0.022 to 0.274)	.10	.062 (–0.073 to 0.231)		.31
Subjective cognitive decline	−.033 (–0.309 to 0.170)	.57	−.034 (–0.319 to 0.165)		.53

^a^β values are standardized regression coefficients.

^b^Adjusted *R*^2^=0.068.

^c^Adjusted *R*^2^=0.118.

### Testing Hypothesis 3: Hierarchical Multiple Regression

**Figure 4:**
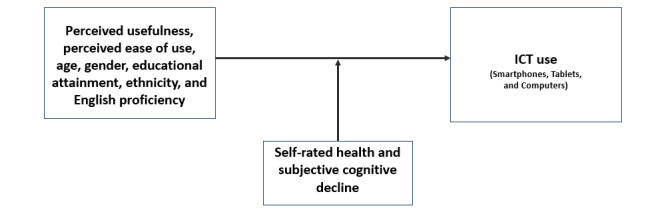
Supported model of information and communications technology (ICT) acceptance among Asian American older adults in low-income housing.

The results of the hierarchical multiple regression are shown in Table 7. In model 1, we found that PU and PEOU were significant predictors of ICT use (PU: β=.560, 95% CI 0.161-0.960; *P*=.01; and PEOU: β=.888, 95% CI 0.506-1.270; *P*<.001). In model 2, adding self-rated health and subjective cognitive decline did not significantly improve model fit for ICT use (Δ*R*^2^=0.005; *P*=.15). In model 3, of the 12 interaction terms that we examined, the interaction between subjective cognitive decline and age was the only one that was statistically significant. Therefore, contrast statements were used to estimate contrasts of interest. Subsequently, we updated the single age variable to reflect 3 different age groups (62 to 74 years, 75 to 84 years, and ≥85 years) and reran the hierarchical multiple regression. In model 3, the interaction terms of subjective cognitive decline and age significantly improved model fit for ICT use (Δ*R*^2^=0.011; *P*=.04). Therefore, our findings support hypothesis 3 (adding self-rated health and subjective cognitive decline as main effects or interaction terms to the TAM will significantly improve model fit), and we reject the null hypothesis. Figure 4 depicts our final supported model of ICT acceptance.

**Table 7:** Hierarchical regression examining our adapted technology acceptance model among older Asian American individuals in low-income housing (N=392)^a^.

Model and independent variable		ICT^b^ use	
		β (SE; 95% CI)	*P* value
**Model 1^c^**			
	Age of 62-74 years	Reference	Reference
	Age of 75-84 years	−.205 (.435; –1.061 to 0.650)	.64
	Age of ≥85 years	−1.215 (.541; –2.280 to –0.150)	.03
	Gender (men)	.423 (.426; –0.415 to 1.261)	.32
	Educational attainment	.539 (.157; 0.230-0.848)	.001
	English proficiency	.579 (.308; –0.027 to 1.186)	.06
	Korean	Reference	Reference
	Chinese	.914 (.519; –0.108 to 1.936)	.08
	Vietnamese	1.238 (.805; –0.347 to 2.823)	.13
	Filipino	−1.250 (1.171; –3.553 to 1.053)	.29
	Asian: other	−1.796 (1.146; –4.051 to .459)	.12
	PU^d^	.560 (.203; 0.161-0.960)	.006
	PEOU^e^	.888 (.194; 0.506-1.270)	<.001
**Model 2 (adding self-rated health and subjective cognitive decline)^f^**			
	Age of 62-74 years	Reference	Reference
	Age of 75-84 years	−.081 (.438; –0.943 to 0.782)	.85
	Age of ≥85 years	−1.063 (.545; –2.136 to 0.010)	.05
	Gender (men)	.397 (.426; –0.441 to 1.235)	.35
	Educational attainment	.525 (.159; 0.212-0.838)	.001
	English proficiency	.390 (.323; –0.247 to 1.026)	.23
	Korean	Reference	Reference
	Chinese	.865 (.519; –0.156 to 1.886)	.10
	Vietnamese	1.323 (.804; –0.260 to 2.906)	.10
	Filipino	−1.324 (1.169; –3.624 to 0.976)	.26
	Asian: other	−1.780 (1.143; –4.029 to 0.469)	.12
	PU	.520 (.204; 0.120-0.921)	.01
	PEOU	.855 (.194; 0.473-1.237)	<.001
	Self-rated health	.374 (.275; –0.166 to 0.915)	.17
	SCD^g^	−.438 (.442; –1.308 to 0.431)	.32
	Δ*R*^2^	0.005	0.15
**Model 3 (adding interaction terms)^h^**			
	Age of 62-74 years	Reference	Reference
	Age of 75-84 years	.352 (.483; –0.599 to 1.303)	.47
	Age of ≥85 years	−.458 (.623; –1.684 to 0.768)	.46
	Gender (men)	.292 (.425; –0.545 to 1.128)	.49
	Educational attainment	.566 (.159; 0.253-0.878)	<.001
	English proficiency	.354 (.321; –0.278 to 0.987)	.27
	Korean	Reference	Reference
	Chinese	.838 (.515; –0.176 to 1.852)	.11
	Vietnamese	1.182 (.802; –0.397 to 2.761)	.14
	Filipino	−1.187 (1.163; –3.474 to 1.101)	.31
	Asian: other	−1.929 (1.136; –4.165 to .306)	.09
	PU	.501 (.202; 0.102-0.899)	.01
	PEOU	.856 (.193; 0.476-1.236)	<.001
	Self-rated health	.356 (.273; –0.181 to 0.893)	.19
	SCD	1.596 (.913; –0.201 to 3.393)	.08
	SCD × age of 62-74 years	Reference	Reference
	SCD × age of 75-84 years	−2.469 (1.074; –4.582 to –.357)	.02
	SCD × age of ≥85 years	−2.920 (1.244; –5.367 to –.473)	.02
	Δ*R*^2^	0.011	0.04

^a^β values are unstandardized regression coefficients.

^b^ICT: information and communications technology.

^c^Adjusted *R*^2^=0.216.

^d^PU: perceived usefulness.

^e^PEOU: perceived ease of use.

^f^Adjusted *R*^2^=0.221.

^g^SCD: subjective cognitive decline.

^h^Adjusted *R*^2^=0.232.

### Subanalysis of Interaction Between Subjective Cognitive Decline and Age

**Figure 5:**
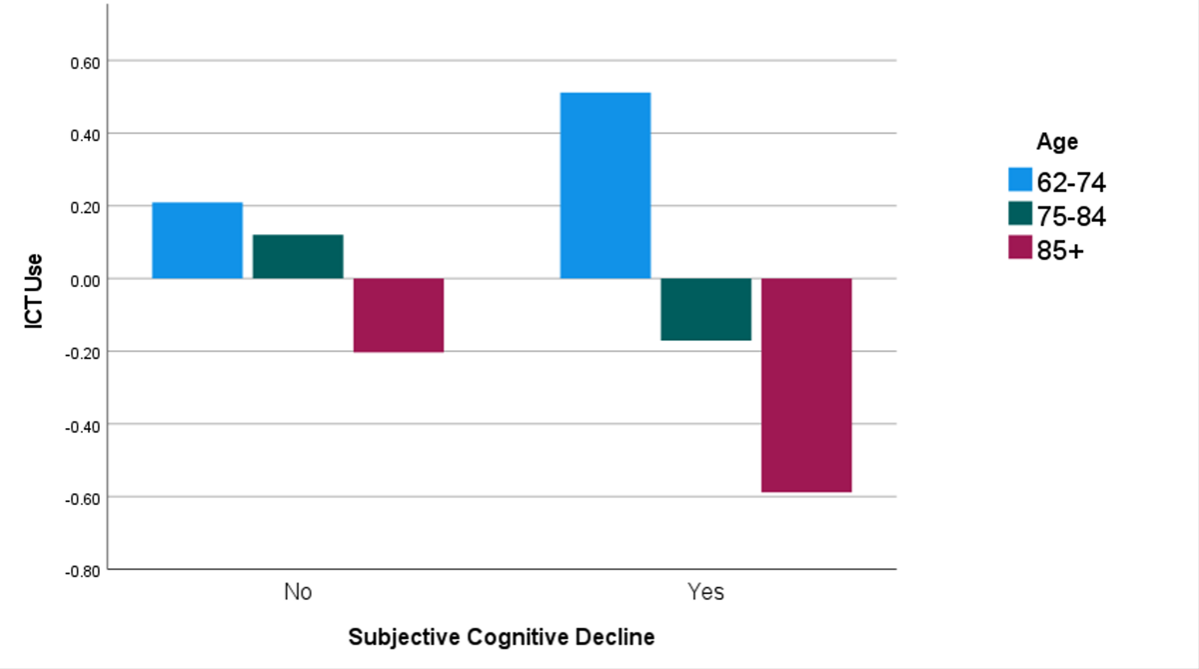
Association between information and communications technology (ICT) use and subjective cognitive decline by age group among Asian American older adults in low-income housing.

The relationship between subjective cognitive decline and ICT use varied in 3 age segments (Figure 5). In the youngest age group (62 to 74 years), ICT use increased with the presence of subjective cognitive decline, whereas in the older age groups (75 to 84 years and ≥85 years), ICT use decreased with the presence of subjective cognitive decline, more so in the oldest age category.

Using regression analysis, we determined that, among participants with subjective cognitive decline, ICT use significantly decreased in the middle and older age groups as compared to the youngest age group. However, among participants without subjective cognitive decline, the difference in use among age groups was not significant (Table 8).

**Table 8:** Contrasts estimating differences in information and communications technology (ICT) use among age categories with and without subjective cognitive decline^a^.

			ICT use		
			Estimate (95% CI)		*P* value
**With subjective cognitive decline**					
	Age of 75-84 years vs age of 62-74 years	−2.509 (–4.906 to –0.111)		.04	
	Age of ≥85 years vs age of 62-74 years	−3.852 (–6.306 to –1.399)		.002	
**Without subjective cognitive decline**					
	Age of 75-84 years vs age of 62-74 years	0.022 (–0.939 to 0.984)		.96	
	Age of ≥85 years vs age of 62-74 years	−0.755 (–2.010 to 0.499)		.24	

^a^Model adjusted for gender, educational attainment, English proficiency, ethnicity, and self-rated health.

## Discussion

### Principal Findings

#### Overview

In this study, we aimed to investigate the relationship among self-rated health, ICT use, PU, and PEOU in a sample of low-income, Asian American older adults living in affordable housing for older adults. We also explored whether the addition of health variables (self-rated health and subjective cognitive decline) would improve TAM model fit. We discovered that the interaction between age and subjective cognitive decline significantly predicted ICT use, which prompted further subanalyses to better understand the direction and strength of these relationships.

#### Self-Rated Health Among Low-Income, Asian American Older Adults

Most of our participants (270/392, 68.9%) reported fair or poor health; this prevalence is nearly 3 times greater than that of all adults aged ≥65 years in California (23.5%) and more than double the prevalence among older Asian Californians (33.1%) [34]. However, according to the CHIS, among older Asian Californians living at or below the federal poverty line, the prevalence of fair or poor health mirrors that of our study population. In 2020 and 2021, a total of 56.9% of Asian American older adults and 75.3% of Korean, 63.7% of Vietnamese, and 51.3% of Chinese individuals aged ≥65 years in the low-income bracket in California reported fair or poor health [34].

In our study population, correlational analysis revealed that older age, lower educational attainment, LEP, and subjective cognitive decline were each significantly and positively associated with poor self-rated health. These findings are consistent with those of other larger studies of older adults in both China and the United States [18,35-37].

#### Associations Among Self-Rated Health, PU, PEOU, and ICT Use

In correlational analysis, self-rated health was also significantly positively associated with PU, PEOU, and ICT use (Table 4). However, significance was not observed when we adjusted for demographic and health factors (Tables 5-7). These findings indicate that factors outside of self-rated health may be greater drivers of technology attitudes and use. Our results are consistent with the findings of the cross-sectional survey by Werner et al [12] involving ethnically diverse, low-income adults aged ≥60 years in California, observing a significant positive association between general health and computer use. However, this relationship lost significance when adjustments were made for demographic and health factors [12]. Initial correlation analyses in other studies also revealed positive associations between self-rated health and ICT use, but these associations became mixed or even reversed directions (eg, changed to a negative association) when adjusting for a variety of covariates, including demographic factors; health factors; and TAM constructs such as PU, PEOU, relevance, and attitude [13,15,36].

#### Significant Interaction Among Age, Subjective Cognitive Decline, and Technology Use

Despite these findings, adding health covariates significantly improved the TAM’s ability to predict ICT use. In particular, the interaction between subjective cognitive decline and age significantly improved model fit. This interaction emerged as an important aspect of our analysis.

Our findings for the youngest age group contradict those of prevailing studies that indicate a negative association between cognitive decline and ICT use [38-41]. In previous studies, mild cognitive impairment has been associated with greater computer-related challenges, reduced frequency and duration of computer use, less accurate mouse use, and longer pauses between mouse movements [39,40].

One possible explanation for our findings is that younger participants, having more lifetime technology exposure, found using ICT devices less cognitively challenging compared to older participants with less previous technology exposure [42-44]. Therefore, the presence of subjective cognitive decline among younger participants may have less of an impact on their actual ICT use. There is evidence suggesting that younger individuals with dementia or mild cognitive impairment have more positive attitudes toward technology as compared to their older counterparts [45].

In addition, it is possible that younger participants with subjective cognitive decline may use ICTs to compensate for cognitive changes. The compensatory use of ICTs is a growing area of research, with a recent systematic review indicating that ICTs can effectively improve prospective memory, task execution, and time management among individuals with impaired cognition [46].

Our contrast analysis indicated that ICT use significantly decreased with age among participants with subjective cognitive decline but not among those without subjective cognitive decline. Our dataset did not indicate the level of severity of cognitive decline, although it is possible that older participants that reported subjective cognitive decline had worse cognition than younger participants who reported subjective cognitive decline. All participants lived independently, although staff at their housing communities described observing cognitive decline among some of the oldest residents. On the basis of previous studies [38-41], it is plausible that older participants with worse cognition would use ICTs less as compared to younger participants with less severe cognitive changes.

As our study was cross-sectional, we cannot determine whether decreasing cognition led to less ICT use or whether more frequent ICT use led to a decrease in cognitive decline. Longitudinal studies suggest that ICT use reduces cognitive decline over time or even improves cognitive function [47], including executive function [48], memory performance [38,48], and the ability to switch between tasks [49]. One study even identified positive associations between daily computer use, hippocampal volume, and memory and executive function [41]. In addition, intervention studies suggest that training older adults to use tablet computers can improve cognitive capacities such as processing speed and episodic memory [50,51].

Of note, within our study population, subjective cognitive decline was significantly associated with English proficiency. In addition, both subjective cognitive decline and English proficiency were significantly associated with age, ICT use, and PEOU. While few studies have delved into the association between subjective cognitive decline and LEP, one study suggests that older Chinese American individuals with LEP are more likely to report subjective cognitive decline than their English-speaking counterparts [52]. Further research is needed to understand the intricate relationships among age, subjective cognitive decline, English proficiency, and ICT use. Cognitive decline experienced concurrently with language barriers may pose unique challenges to technology use among older adults.

#### Complexity of the Relationship Between Health and ICT Use

The relationship between health and ICT use is complex. Cognitive abilities or physical health can be both a precursor and outcome of ICT use. Increasingly, ICTs modulate access to health care, services, information, and social interaction, thereby influencing overall health outcomes. Individuals who do not use ICTs face limitations in accessing telemedicine, web-based prescription refills, web-based health information, and web-based management of Medicare and other types of health insurance. Moreover, ICT use may have adverse effects on health, particularly if it exposes older adults to scams, fraud, or identity theft. The internet offers unlimited access to information—some of it reliable, but much of it being false. In cases in which individuals struggle to discern trustworthy information, the internet can become a source of confusion or, even worse, misinformation.

### Study Limitations

Our study has several limitations. The data represent a specific population of Asian American older adults; most participants were Korean, and all participants lived in an age-limited setting (affordable housing for older adults), limiting the generalizability of our findings. However, few studies exist targeting this significant Asian population group, enhancing within-group understanding. The use of self-reported measures, including self-rated health and subjective cognitive decline, could introduce response bias.

Due to low educational attainment among study participants, validated survey measures were shortened and adapted to the population. In this study, we operationalized TAM constructs based on elements analogous to the original TAM measures. Nevertheless, the comparability of results across studies is challenging due to variations in outcomes, the operationalization of constructs, and the inclusion of diverse covariates in regression analyses.

Despite these limitations, our sample was unique in that all participants were of low income and represented various Asian ethnicities and most participants had LEP. Our findings have implications for promoting digital engagement among low-income Asian American older adults. Our findings indicate that health-related factors, particularly subjective cognitive decline, can play a role in shaping technology adoption and use patterns. Tailored interventions and support strategies should consider the age-related nuances in the relationship between cognitive decline and technology use.

Enhancing factors such as PEOU and PU through digital literacy training and user interface customization can be particularly beneficial for increasing ICT acceptance among this population [53-58]. Demonstrating culturally relevant use cases for technology, such as connecting with overseas relatives, accessing health information, or enjoying entertainment in one’s native language, can make technology more meaningful for Asian American older adults. For example, the Lighthouse for Older Adults program, which provided the data for this study, improved technology adoption in affordable housing for older adults by offering equipment, training, and support, thereby fostering a community of learners who benefit from shared experiences [59].

The study population had very low English proficiency, and most technology uses Roman characters and is English based. Addressing language barriers is crucial for promoting ICT acceptance and use among low-income, Asian American older adults. Expanding the language capabilities of voice interfaces such as Google Voice and Alexa can make these technologies more user-friendly for those who do not write, read, or speak English [60,61].

Furthermore, the costs of internet access and devices can hinder ICT use among low-income older adults. During the COVID-19 pandemic, several policies were implemented to address these financial barriers. The Accessible, Affordable Internet for All Act of 2021 allocated US $94 billion for broadband infrastructure and subsidies [62]; the Infrastructure Investment and Jobs Act of 2021 set aside US $65 billion for internet access, digital literacy, and rural expansion [63]; and the Affordable Connectivity Program offered low-income families a US $30 monthly subsidy toward their internet costs and required internet service providers to be transparent about pricing [63]. Although the Affordable Connectivity Program ended in February 2024, the Lifeline support program now offers families a smaller subsidy. New state programs are emerging, but continued advocacy is necessary to improve the affordability of digital access.

Future research could further explore the reasons behind the age-dependent effects of cognitive decline on technology use and examine potential interventions to enhance technology accessibility as well as digital literacy and technology adoption among older adults with varying health profiles. Longitudinal studies and intervention designs could provide a deeper understanding of these dynamics.

### Conclusions

This study contributes to our understanding of the intricate relationship among self-rated health, subjective cognitive decline, and the acceptance and use of ICTs among low-income, Asian American older adults. By highlighting the age-specific effects of cognitive decline on technology adoption, our findings can inform targeted interventions to promote digital engagement and enhance the quality of life of aging populations.

## Abbreviations

CHIS: California Health Interview Survey
ICT: information and communications technology
LEP: limited English proficiency
PEOU: perceived ease of use
PU: perceived usefulness
TAM: technology acceptance model

## Data Availability

The datasets generated during and analyzed during this study are available from the corresponding author on reasonable request.
